# Secondary reconstruction with a transverse colon covered with a pectoralis major muscle flap and split thickness skin grafts for an esophageal defect and wide skin defects of the anterior chest wall

**DOI:** 10.1186/s40792-015-0020-x

**Published:** 2015-02-24

**Authors:** Noriaki Sadanaga, Keigo Morinaga, Hiroshi Matsuura

**Affiliations:** Department of Surgery, Saiseikai Fukuoka General Hospital, 1-3-46 Tenjin, Chuo-ku, Fukuoka 810-0001 Japan; Department of Plastic Surgery, Saiseikai Fukuoka General Hospital, 1-3-46 Tenjin, Chuo-ku, Fukuoka 810-0001 Japan

**Keywords:** Esophageal defect, Secondary reconstruction, Pectoralis major muscle flap, Split thickness skin grafts

## Abstract

Necrosis of a reconstructed organ after esophagectomy is a rare postoperative complication. However, in case this complication develops, severe infectious complications can occur, and subsequent surgical reconstruction is quite complicated. To treat esophageal conduit necrosis after esophageal reconstruction with the terminal ileum and ascending colon, we reconstructed the esophagus using a transverse colon, which was covered with a pectoralis major muscle flap to reinforce the anastomotic site. In addition, split thickness skin grafts were applied to the wide skin defect to cover the reconstructed organs at the antesternal route. Widely extended split thickness skin grafts can adhere to the reconstructed organs without excessive tension. Therefore, this method enabled successful treatment of an esophageal defect and wide skin defects of the anterior chest wall.

## Background

Patients with thoracic esophageal and gastric cancer especially those with advanced cancer, usually undergo subtotal esophagectomy and total gastrectomy and reconstruction using the colon [1-4]. The antesternal route (subcutaneous route) rather than the intrathoracic or retrosternal route of reconstruction is indicated in these cases, because colon interposition is considered a high-risk operation for anastomosis [5]. According to a summary of previously reported data, the incidence of anastomotic leakage after colonic interposition ranged from 0% to 46%, and that of colon necrosis ranged from 0% to 11.8% [2]. Once necrosis of the esophageal conduit occurs, the necrotic tissue must be resected, and the proximal esophagus must be diverted [6]. Moreover, an inflammatory and necrotic change often occurs in the skin and subcutaneous tissue of the anterior chest wall, and debridement of the necrotic tissues is also required [7]. Therefore, secondary reconstruction options for esophageal discontinuity are considerably limited and quite complicated [8].

We describe secondary esophageal reconstruction using a transverse colon that was covered with a pectoralis major muscle flap and split thickness skin grafts in a patient with an esophageal defect and wide skin defect; this procedure was performed to treat necrosis of the esophageal conduit after esophageal reconstruction with the terminal ileum and ascending colon.

## Case presentation

A 58-year-old woman underwent surgery for thoracic esophageal cancer (T3N2M0: Stage IIIB) and gastric cancer (T3N0M0: Stage IIB). Under satisfactory general anesthesia with the patient in a left lateral position, thoracotomy was performed through the fourth intercostal space, and we performed subtotal esophagectomy with lymph node dissection. The patient’s position was changed to supine, and the abdomen was opened by an upper midline incision. Total gastrectomy was performed; the right colon and terminal ileum were mobilized, dividing the ascending colon at the hepatic flexure and dividing the ileum at the 10-cm oral side from the ileum end. After the ileocolic artery and vein were divided, the right colon with terminal ileum was moved to the neck via the antesternal route. The cervical esophagus was anastomosed to the ileum, and the ascending colon was anastomosed to the Roux-en-Y loop of the jejunum.

**Figure 1:**
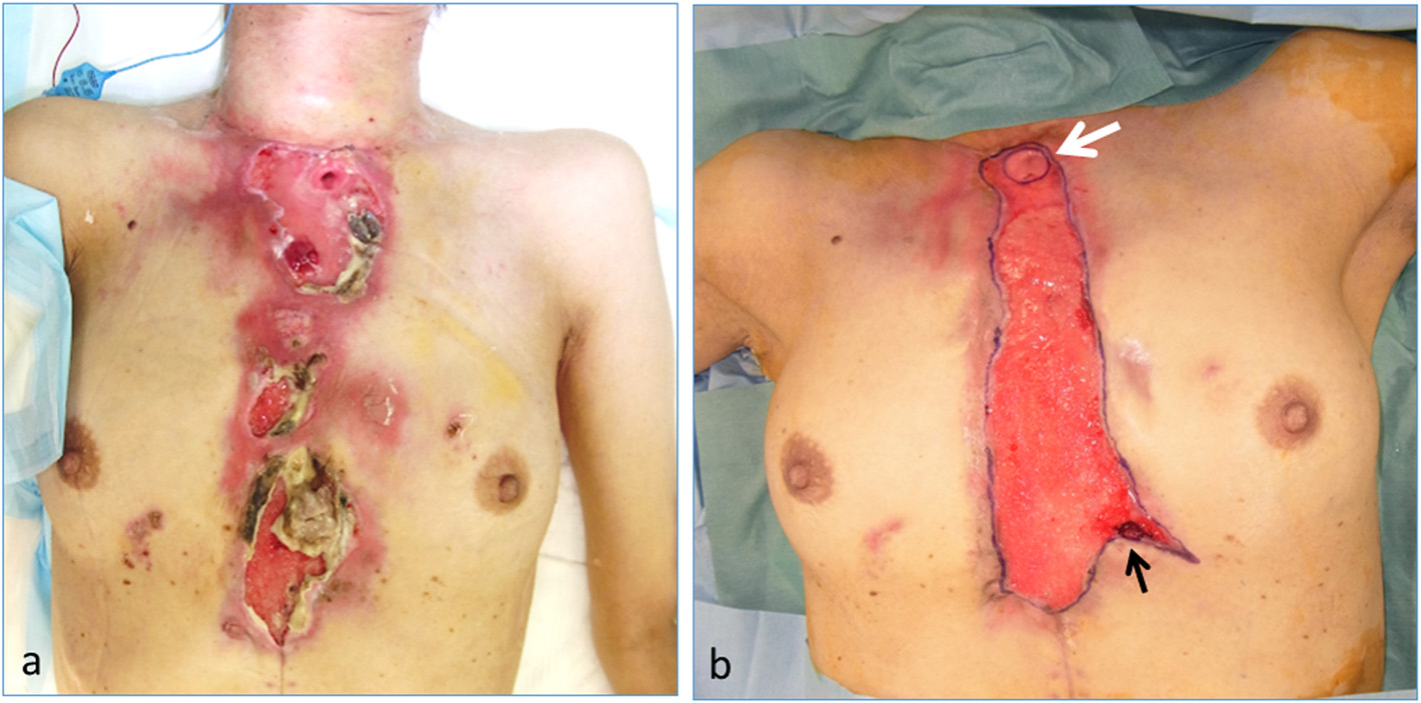
**Necrosis of the esophageal conduit after esophageal reconstruction with the terminal ileum and ascending colon. (a)** Necrosis of the terminal ileum and skin of the anterior chest wall. **(b)** Post-debridement and infection control. The esophageal defect (esophagostomy (white arrow) and the distal end of the resected colon (black arrow)) and the wide skin defect of the anterior chest wall (20 × 7 cm).

The anastomosis leaked in the cervical esophagus and ileum on postoperative day 5, and subsequently, necrosis of the terminal ileum and infection of the anterior chest wall occurred (Figure 1a). Thus, debridement of the reconstructed organ (the terminal ileum and ascending colon) and infected skin of the anterior chest wall was performed. Esophagostomy was also performed, and the non-necrotic distal end of the colon was closed. Continuous irrigation and drainage sufficiently controlled the infection of the anterior chest wall. However, there was a wide skin defect of the anterior chest wall (Figure 1b).

**Figure 2:**
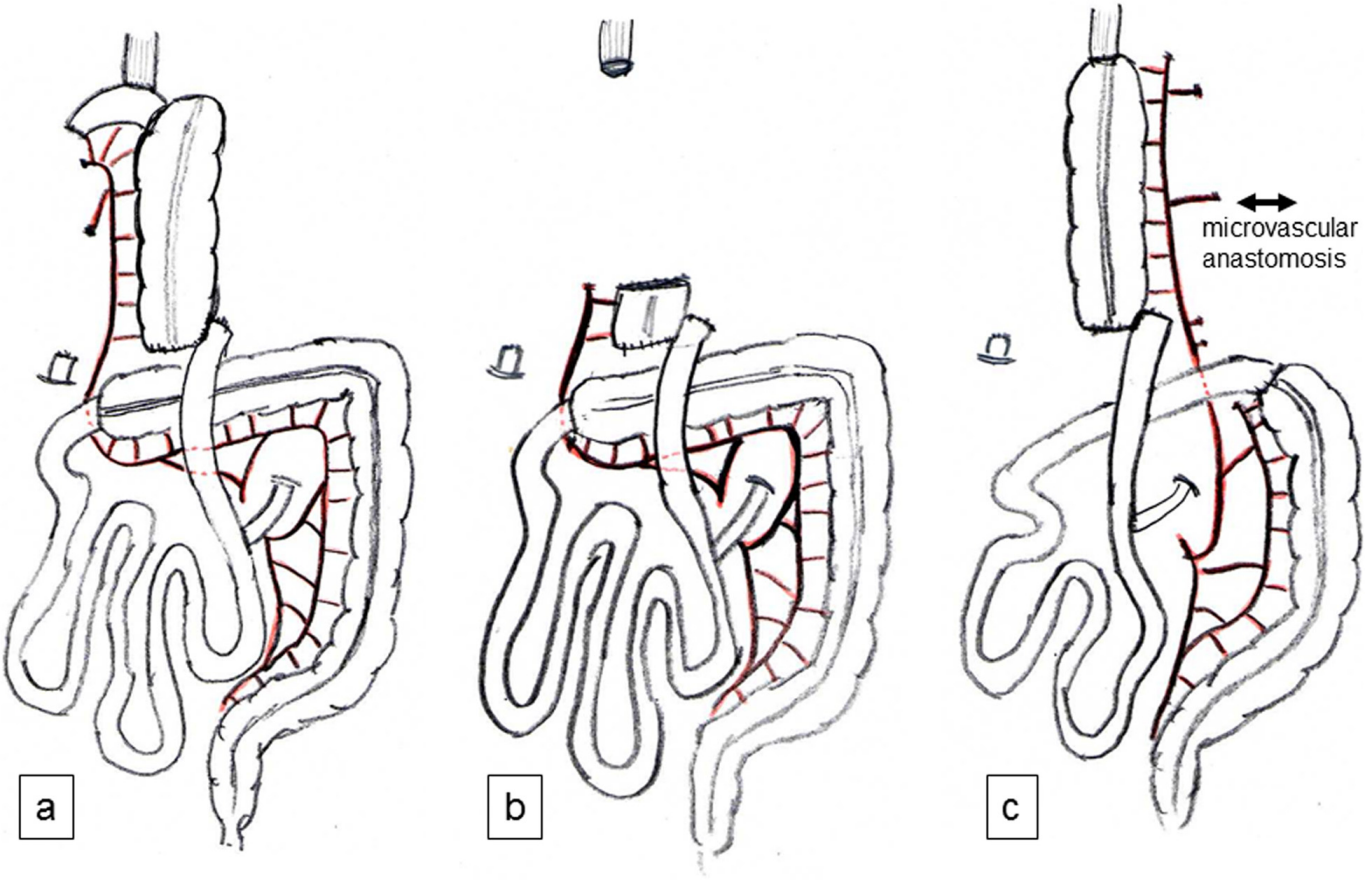
**Schema of the reconstructed esophageal conduit. (a)** The first reconstruction. The right side colon (the terminal ileum and the ascending colon) which was supplied by the marginal artery from the middle colic artery. **(b)** The resection of the necrotic reconstructed organ (the terminal ileum and part of the ascending colon). **(c)** The second reconstruction. The transverse colon which was supplied by the marginal artery from the left colic artery, and the microvascular anastomosis of the left internal thoracic artery and vein to the left branch of the middle colic artery and vein.

**Figure 3:**
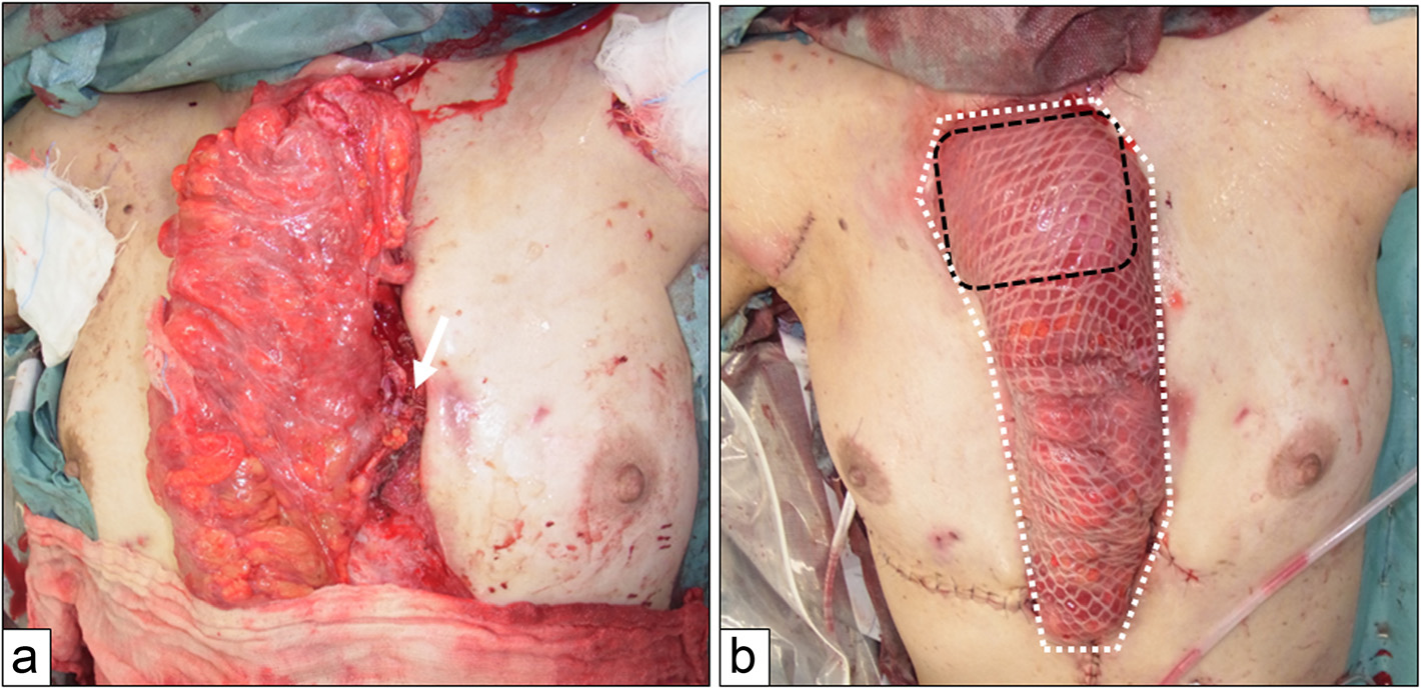
**The second reconstruction using the transverse colon. (a)** The microvascular anastomosis of the left internal thoracic artery and vein was added to the left branch of the middle colic artery and vein (white arrow). **(b)** Anastomosis of the cervical esophagus and transjverse colon was reinforced using the right pectoralis major muscle flap (black dot), and the transverse colon and wide skin defect of the anterior chest wall were covered with split thickness skin grafts (white dot).

**Figure 4:**
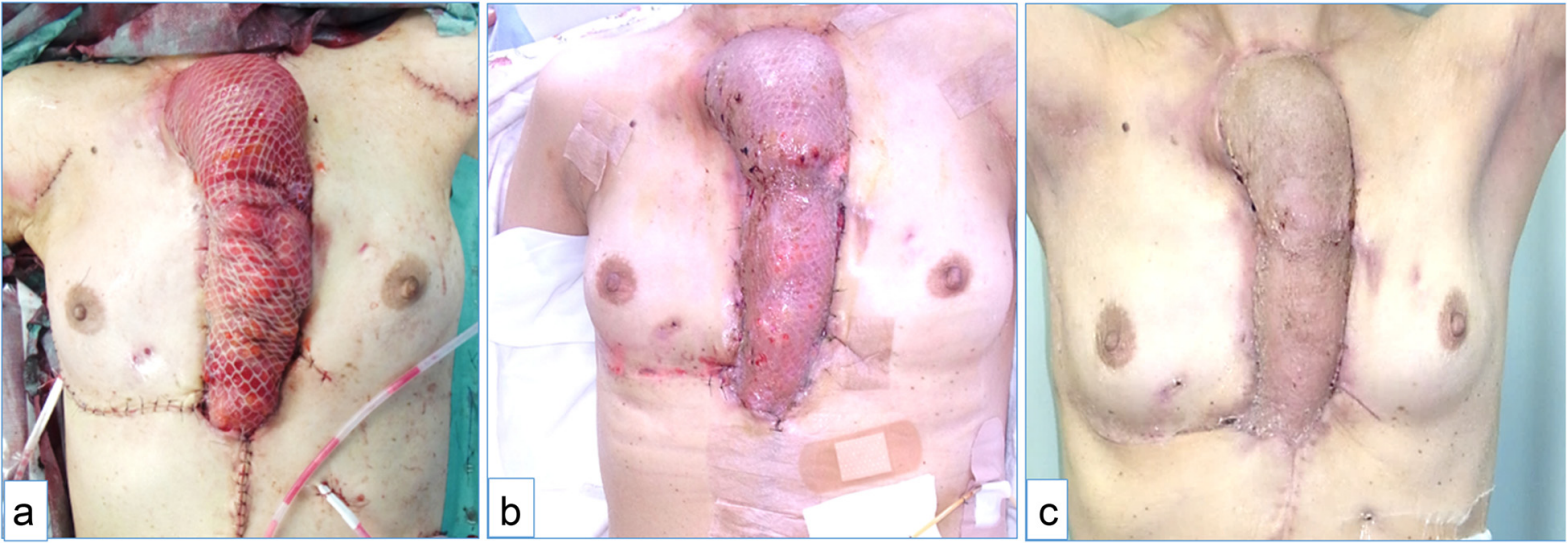
**Esophageal conduit covered with a pectoralis major muscle flap and split thickness skin graft. (a)** A transverse colon covered with a pectoralis major muscle flap and split thickness skin grafts. **(b)** Split thickness skin grafts adhering to the reconstructed organs (postoperative day 10). **(c)** Split thickness skin grafts requiring no dressing materials (postoperative day 27).

Finally, we performed re-reconstruction of the esophagus (Figure 2). The transverse colon, which was supplied by the left colic artery, was used for organ reconstruction, and the microvascular anastomosis of the left internal thoracic artery and vein was added to the left branch of the middle colic artery and vein (Figure 3a). The cervical esophagus was anastomosed to the proximal transverse colon, and the distal transverse colon was anastomosed to the Roux-en-Y loop of the jejunum. The anastomosis of the cervical esophagus and transverse colon was covered with the right pectoralis major muscle flap, which preserved the pectoral branch from the thoracoacromial artery. For the wide range of skin defects of the anterior chest wall, split thickness skin grafts from the left thigh using a dermatome were implanted to cover the reconstructed organs and wide skin defect (Figures 3b and 4a).

The patient’s postoperative course was without any serious complications. Split thickness skin grafts adhered to the reconstructed organ tissues 10 days postoperatively (Figure 4b). On postoperative day 14, the patient resumed oral intake after confirming the absence of abnormal leakage or stenosis on the esophagogram. On postoperative day 27, the patient was able to eat enough food and was discharged (Figure 4c).

## Discussion

Secondary reconstruction of a failed esophageal reconstruction is quite difficult [6-9]. For secondary reconstruction of the esophagus, several organs are used, including the jejunal interposition, free jejunum, and skin roll prepared from a myocutaneous flap as well as the colon interposition [7]. Among these, colon tissue can be used for the long segment replacement. In our case, the right colon segment (the terminal ileum to the ascending colon), which was supplied by the middle colic artery, was used as the primary reconstruction organ. Therefore, the transverse colon, which was supplied by the left colic artery, was used antegradely as the secondary reconstruction organ.

The necessity of additional microvascular anastomosis is controversial. Mine et al. reported that it is unnecessary to routinely use microvascular surgery during colon reconstruction, because no graft necrosis has been observed; thus, 96.5% of their patients underwent colon reconstruction without microvessel anastomosis [4]. In contrast, Saeki et al. demonstrated that colon interposition with microvascular surgery, especially superdrainage, was associated with satisfactory postoperative outcomes, including no serious anastomotic problems [2]. In our case, additional microvascular anastomosis was not performed in the first esophageal reconstruction. However, in the second reconstruction, microvascular anastomosis was performed in the artery and vein to achieve a more reliable postoperative outcome. Therefore, microvascular anastomosis should be considered after colonic interposition, because it is possible to prevent colon necrosis during insufficient blood circulation.

The pectoralis major muscle flap is widely used for various purposes, especially in head and neck reconstruction surgery. The use of the pectoralis major muscle flap involves a well-established technique, and because it is well vascularized, it can be easily mobilized. Heitmiller et al. reported the use of the pectoralis major myocutaneous flap in the management of cervical esophageal anastomotic complications [10]. Additionally, Morita et al. reported that in patients with esophageal cancer, the simple method of using the pectoralis major muscle flap to cover the anastomotic repair site prevents the development of recurrent leakage after reconstruction via the subcutaneous route [9]. Similarly, we applied a pectoralis major muscle flap to reinforce the anastomotic site.

Split thickness skin grafts can be meshed by cutting slits into the sheet of a graft and expanding it. Meshed grafts are quite useful when there is a paucity of available donor skin, the recipient bed is bumpy or convoluted, or the recipient bed is suboptimal as with exudate. Split thickness skin grafts readily take to the recipient site, and the donor site re-epithelializes quickly [11]. Split thickness skin grafts have also been used to cover the abdominal viscera in patients with a wide abdominal wall defect during planned hernia repair [12]. In our case, split thickness skin grafts were used to cover the reconstructed organ (the transverse colon) with a wide skin defect of the anterior chest wall. In addition, a wide range of skin defects can be repaired using split thickness skin grafts without excessive tension of the reconstructed organ.

Okazaki et al. also reported that because primary wound closure is often difficult in the secondary reconstruction of the esophagus, the pectoralis major musculocutaneous flap is reliable for covering the reconstructed esophagus. In those cases, they reported that a pectoralis major musculocutaneous flap was used to cover the reconstructed esophagus, and the flap donor site of the anterior chest wall was repaired with a split thickness skin graft [7]. In our method, the anastomotic site was fully covered with the pectoralis major muscle flap, and the esophageal conduit was covered with a split thickness skin graft. Since this causes less deformation to the anterior chest wall and the bilateral breast, this method should be considered in female patients because of the cosmetic advantage.

## Conclusions

In conclusion, we propose performing esophageal reconstruction with the new reconstructed organ covered with the pectoralis major muscle flap and split thickness skin grafts for secondary reconstruction after necrosis of the esophageal conduit and a wide skin defect of the anterior chest wall.

## Consent

Written informed consent was obtained from the patient for publication of this Case report and any accompanying images.
